# Surface refraction of sound waves affects calibration of three-dimensional ultrasound

**DOI:** 10.1186/s13014-015-0424-6

**Published:** 2015-05-27

**Authors:** Hendrik Ballhausen, Bianca Désirée Ballhausen, Martin Lachaine, Minglun Li, Katia Parodi, Claus Belka, Michael Reiner

**Affiliations:** Department of Radiation Oncology, University Hospital of Ludwig-Maximilians-University, Marchioninistraße 15, 81377 Munich, Germany; Small Animal Clinic Haar, Keferloher Strasse 25, 85540 Haar, Germany; Elekta Ltd, 2050 de Bleury, Montréal, QC H3A2J5, Canada; Department of Experimental Physics, Medical Physics, Ludwig-Maximilians-University, Am Coulombwall 1, 85748 Garching, Germany

## Abstract

**Background:**

Three-dimensional ultrasound (3D-US) is used in planning and treatment during external beam radiotherapy. The accuracy of the technique depends not only on the achievable image quality in clinical routine, but also on technical limitations of achievable precision during calibration. Refraction of ultrasound waves is a known source for geometric distortion, but such an effect was not expected in homogenous calibration phantoms. However, in this paper we demonstrate that the discontinuity of the refraction index at the phantom surface may affect the calibration unless the ultrasound probe is perfectly perpendicular to the phantom.

**Methods:**

A calibration phantom was repeatedly scanned with a 3D-US system (Elekta Clarity) by three independent observers. The ultrasound probe was moved horizontally at a fixed angle in the sagittal plane. The resulting wedge shaped volume between probe and phantom was filled with water to couple in the ultrasound waves. Because the speed of sound in water was smaller than the speed of sound in Zerdine, the main component of the phantom, the angle of the ultrasound waves inside the phantom increased. This caused an apparent shift in the calibration features which was recorded as a function of the impeding angle. To confirm the magnitude and temperature dependence, the experiment was repeated by two of the observers with a mixture of ice and water at 0 °C and with thermalized tap water at 21 °C room temperature.

**Results:**

During the first series of measurements, a linear dependency of the displacements dx of the calibration features on the angle α of the ultrasound probe was observed. The three observers recorded significantly nonzero (p < 0.0001) and very consistent slopes of dx/dα of 0.12, 0.12, and 0.13 mm/°, respectively..

At 0 °C water temperature, the slope increased to 0.18 ± 0.04 mm/°. This matched the prediction of Snell’s law of 0.185 mm/° for a speed of sound of 1,402 m/s at the melting point of ice.

At 21 °C, slopes of 0.11 and 0.12 mm/° were recorded in agreement with the first experiment at about room temperature. The difference to the theoretical expectation of 0.07 mm/° was not significant (p = 0.09).

**Conclusions:**

The surface refraction of sound waves my affect the calibration of three-dimensional ultrasound. The temperature dependence of the effect rules out alternative explanations for the observed shifts in calibration. At room temperature and for a structure that is 10 cm below the water-phantom interface, a tilt of the ultrasound probe of 10° may result in a position reading that is off by more than half a millimeter. Such errors are of the order of other relevant errors typically encountered during the calibration of a 3D-US system. Hence, care must be taken not to tilt the ultrasound probe during calibration.

## Background

In image guided radiotherapy, the ability to improve tumor control and reduce toxicity [1], e.g. through dose escalation and hypo-fractionation [2], and through advanced techniques [3, 4], relies on an accurate target concept [5] and precise positioning to achieve the desired dose distribution [6, 7].

Three-dimensional ultrasound (3D-US) is an attractive imaging modality in external beam radiotherapy [8, 9], both during planning (e.g. to improve soft tissue contrast for better target and organ at risk delineation) and during treatment (for non-invasive dose-free patient positioning control, and monitoring of inter- and intra-fraction organ movements). The accuracy of the technique depends not only on the achievable image quality in clinical routine [10], but also on technical limitations of achievable precision during calibration [11].

In ultrasound imaging, speed-of-sound effects [12, 13] and refractive effects have been known to distort the reconstructed geometry, and iterative approaches have been developed to reverse the curvature of ultrasound waves as described by Snell’s law [14, 15]. Recently, such corrections have been applied to regions of the body known to harbor strong gradients in refraction index, like the cranium [16] and the breast [17].

Calibration phantoms, however, are typically made of homogenous tissue equivalents, and refraction inside the phantom should not affect calibration results. However, in this paper we demonstrate that at the phantom surface a discontinuity in the refraction index will cause refraction of the entering ultrasound waves, whenever the ultrasound probe is not perfectly perpendicular to the surface. The effect is studied and confirmed under real calibration conditions with tap water as coupling element and at room temperature.

## Methods

The experiment consisted of a 3D-US of type Clarity and a calibration phantom, both made by Elekta (Stockholm, Sweden). The ultrasound system was composed of a free-hand US probe, attached to a mobile bedside work-station with touch-screen, and a ceiling-mounted stereoscopic infrared camera, tracking the free-hand probe via infrared reflectors. The calibration phantom was made from tissue equivalent Zerdine. It featured infrared reflectors on the outside (visible to the stereoscopic infrared camera) and a hypoechoic cavity inside (visible to ultrasound). During the experiment, the tip of the ultrasound probe was submerged into water covering the surface of the phantom. The water had the same function as the gel layer commonly used in clinical routine, to couple in the ultrasound waves without reflection. During each measurement, the hypoechoic cavity was scanned and registered, and any deviation from zero was reported by the ultrasound workstation. All scans were repeated ten times to achieve sub-millimeter precision.

**Fig. 1:**
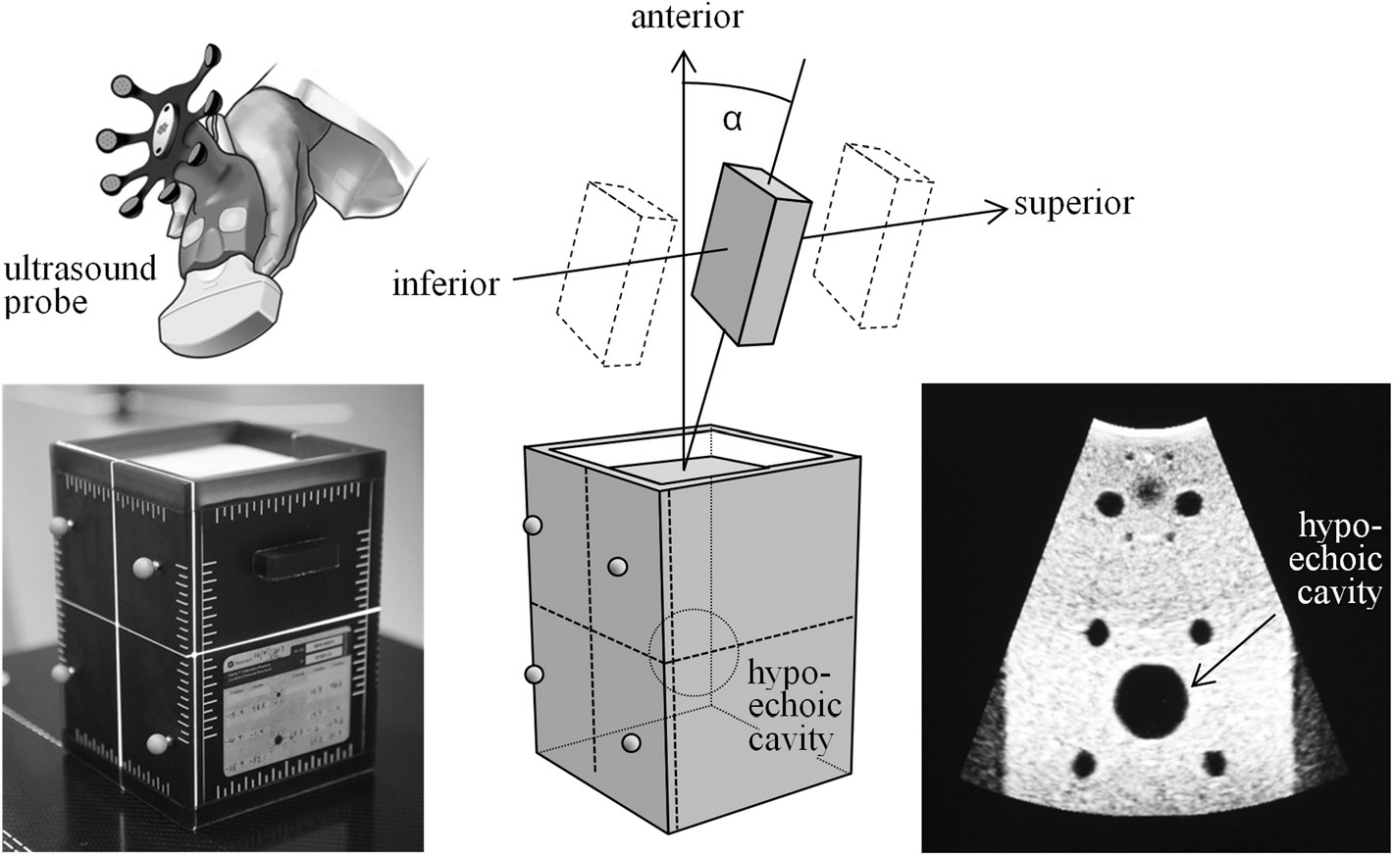
Experimental setup

The experiment is sketched in Fig. 1. The phantom was scanned by moving the ultrasound probe horizontally from inferior to superior. These movements were purely translatory, the ultrasound probe was not rotated during the movement (as would typically be done in clinical routine). Instead, there was a constant angle α between the vertical axis and the axis of the ultrasound probe that was maintained fixed during the movement. This angle was defined as positive α (ultrasound probe tilted within the sagittal plane from posterior/inferior to anterior/superior) or negative α (from ultrasound probe tilted from posterior/superior to anterior/inferior).

This translatory movement at a fixed angle was facilitated by a hand-made cardboard showing lines of -5°, 0°, +5°, +10° which could be attached to the phantom. It was discarded after measurements. It featured lines at the respective angle (s) that were aligned with the axis of the ultrasound probe. The visual and manual uncertainty of setting and keeping the angle during the hand-held measurements was estimated by visual inspection to be ±1°.

First, three independent observers (BH, BBD, RM) each scanned the phantom each ten times at α = 0° (ultrasound probe held straight upright), α = +10°, α = -5°, and α = +5°. Angles beyond this range were not feasible because otherwise the hypoechoic spheroid would not have been completely covered in the scan range. For each of the observers, the recorded longitudinal positions (x-values) of the spheroid were plotted as the dependent variable in a scatter plot against the angle α as independent variable. The slope dx/dα of the line of best fit was calculated from linear regression. The average slope and error margin was calculated from the slopes of the three observers.

**Fig. 2:**
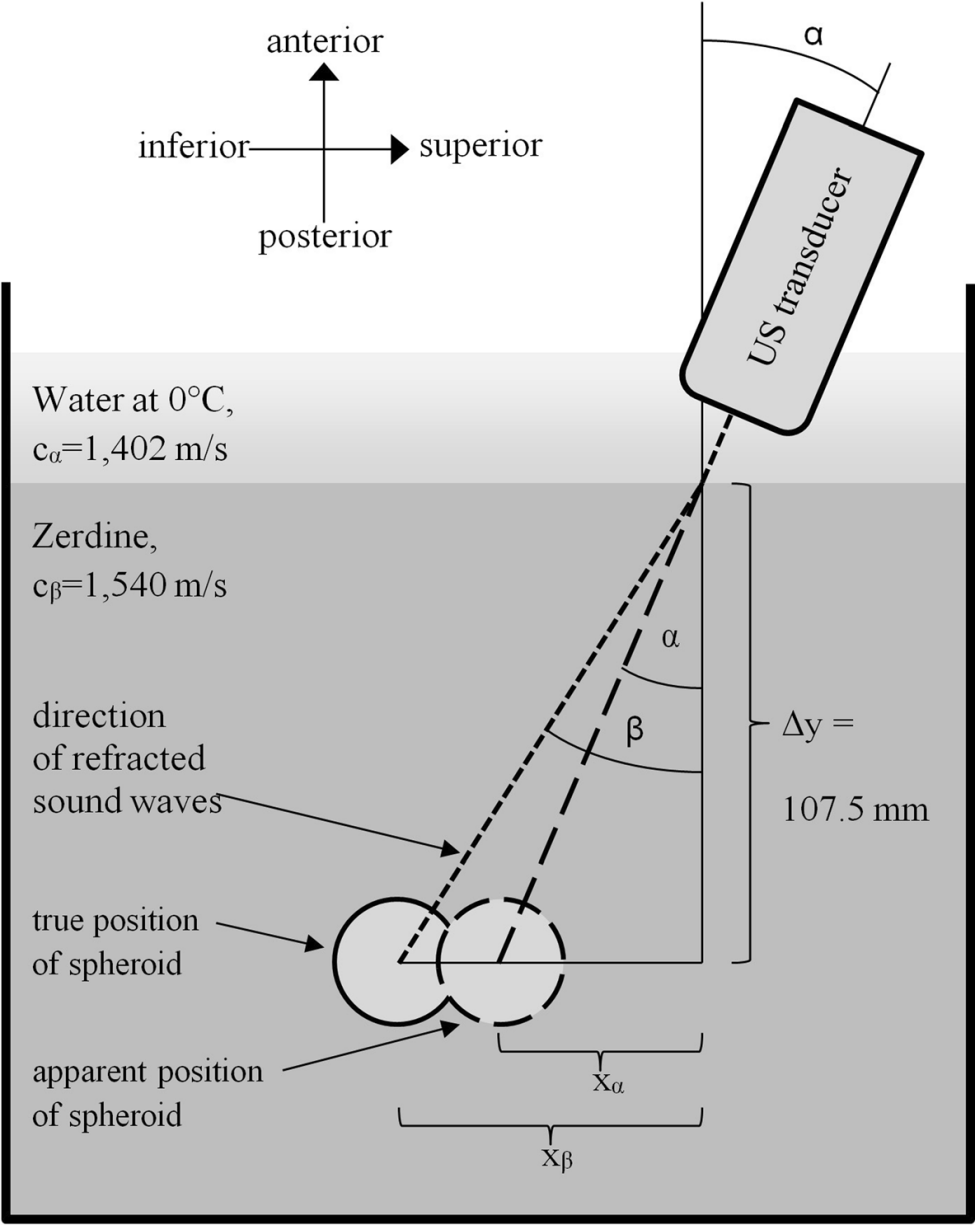
Snell’s law governs the refraction of ultrasound waves at the interface between water and phantom

After reviewing the results of the first experiment, the hypothesis was formed that the observed slope resulted from the refraction of the ultrasound waves at the boundary interface between water and phantom (Zerdine), see Fig. 2. The speed of sound in water (c_α_ = 1,402 m/s at 0 °C) is smaller than in Zerdine (c_β_ = 1,540 m/s reported by the manufacturer). Hence, any nonzero angle α (as measured outside of the phantom) would be enlarged to β (as measured inside of the phantom) by Snell’s law: sin β = sin α · c_β_ / c_α_. As a consequence, the apparent position x_α_ of the spheroid would differ from its true position x_β_ by Δx = Δy · tan α - Δy · tan β. If α varies, so does β and Δx, and the perceived slope is approximately d(Δx/Δy) / dα = c_β_ / c_α_ – 1 (followed by negligible corrections of order α^2^). The depth Δy of the center of the spheroid was measured by a single observer (MR) and the experimental findings were compared with the approximate value expected at room temperature.

Thus, because the speed of sound in water depends on temperature, and because the temperature of water had not been recorded in the first series of measurements, the experiment was repeated with a mixture of ice and water. Melting ice in equilibrium with water has a physically defined temperature of 0 °C with known speed of sound of 1,402 m/s. Two observers (BH, BBD) again recorded each ten measurements at each four angles. Again, linear regression was performed and d(Δx/Δy) / dα calculated and compared to the predicted value of c_β_ / c_α_ – 1. The same two observers finally repeated the experiment at room temperature. The tap water used was given time to thermalize on top of the calibration phantom, and its temperature was recorded over the duration of the two measurement series.

## Results

**Fig. 3:**
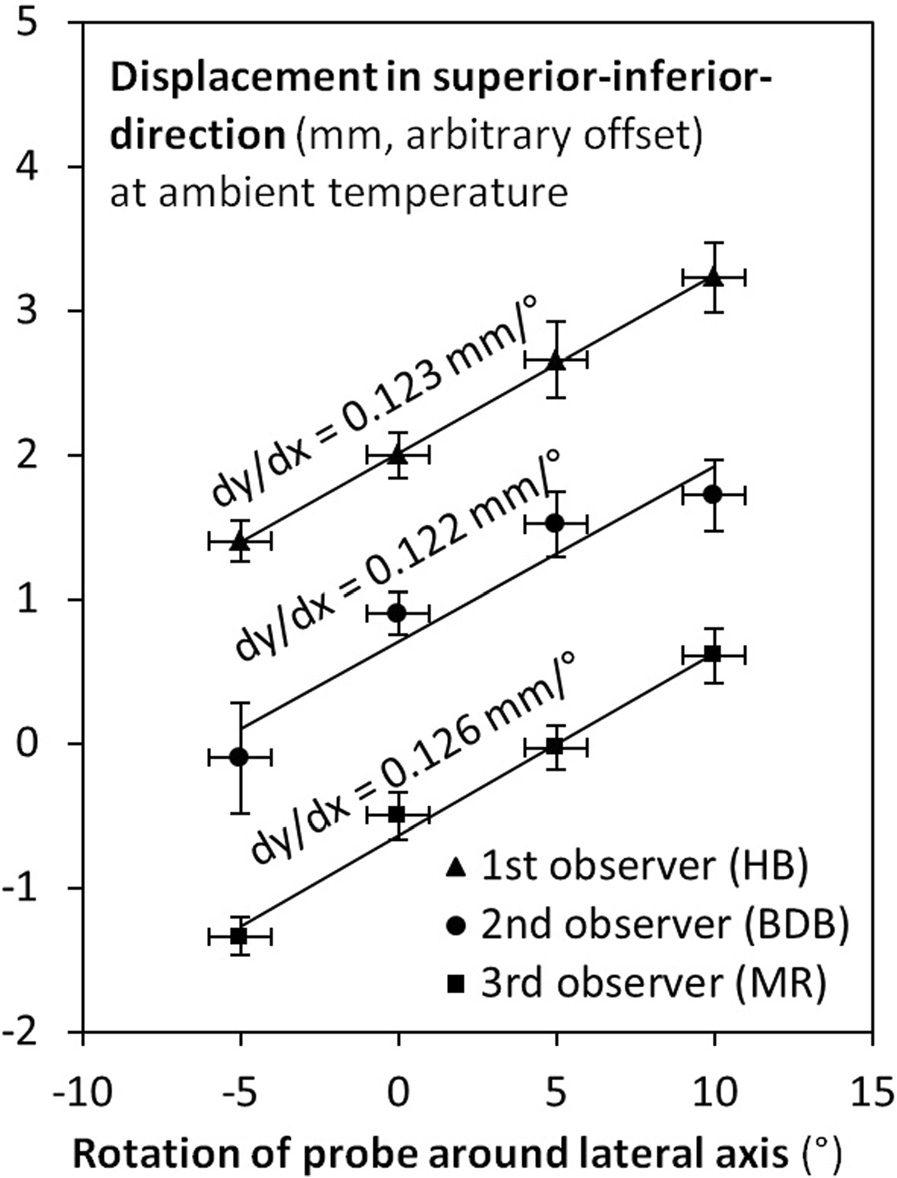
During the first experiment, there was a linear relationship between probe orientation and position reading

During the first series of measurements, the three observers measured slopes dx / dα of 0.12 mm/°, 0.12 mm/°, and 0.13 mm/°, respectively. Given error bounds from regression of less than 0.01 mm/°, these slopes are significantly different from zero, and the (approximately) linear dependency is well visible in the data, see Table 1 and Fig. 3.

**Table 1:** Ultrasound probe orientation affected mostly the longitudinal position readings

Number of experiment, number of observer	Slope in longitudinal direction (mm/°)	Slope in lateral direction (mm/°)	Slope in vertical direction (mm/°)
1^st^ experiment, 1^st^ observer (HB)	+0.123 ± 0.006 (r^2^ = 0.92)	–0.016 ± 0.005 (r^2^ = 0.19)	+0.004 ± 0.002 (r^2^ = 0.06)
1^st^ experiment, 2^nd^ observer (BDB)	+0.122 ± 0.009 (r^2^ = 0.82)	–0.015 ± 0.004 (r^2^ = 0.30)	– 0.010 ± 0.004 (r^2^ = 0.16)
1^st^ experiment, 3^rd^ observer (MR)	+0.126 ± 0.005 (r^2^ = 0.95)	–0.013 ± 0.005 (r^2^ = 0.16)	+0.001 ± 0.003 (r^2^ = 0.00)
2^nd^ experiment, 1^st^ observer (HB)	+0.211 ± 0.009 (r^2^ = 0.93)	–0.017 ± 0.008 (r^2^ = 0.12)	+0.026 ± 0.009 (r^2^ = 0.18)
2^nd^ experiment, 2^nd^ observer (BDB)	+0.157 ± 0.008 (r^2^ = 0.91)	+0.014 ± 0.006 (r^2^ = 0.12)	+0.035 ± 0.006 (r^2^ = 0.43)
3^rd^ experiment, 1^st^ observer (HB)	+0.124 ± 0.006 (r^2^ = 0.93)	+0.013 ± 0.004 (r^2^ = 0.21)	-0.016 ± 0.003 (r^2^ = 0.48)
3^rd^ experiment, 2^nd^ observer (BDB)	+0.110 ± 0.005 (r^2^ = 0.92)	+0.013 ± 0.003 (r^2^ = 0.42)	-0.011 ± 0.003 (r^2^ = 0.29)

**Fig. 4:**
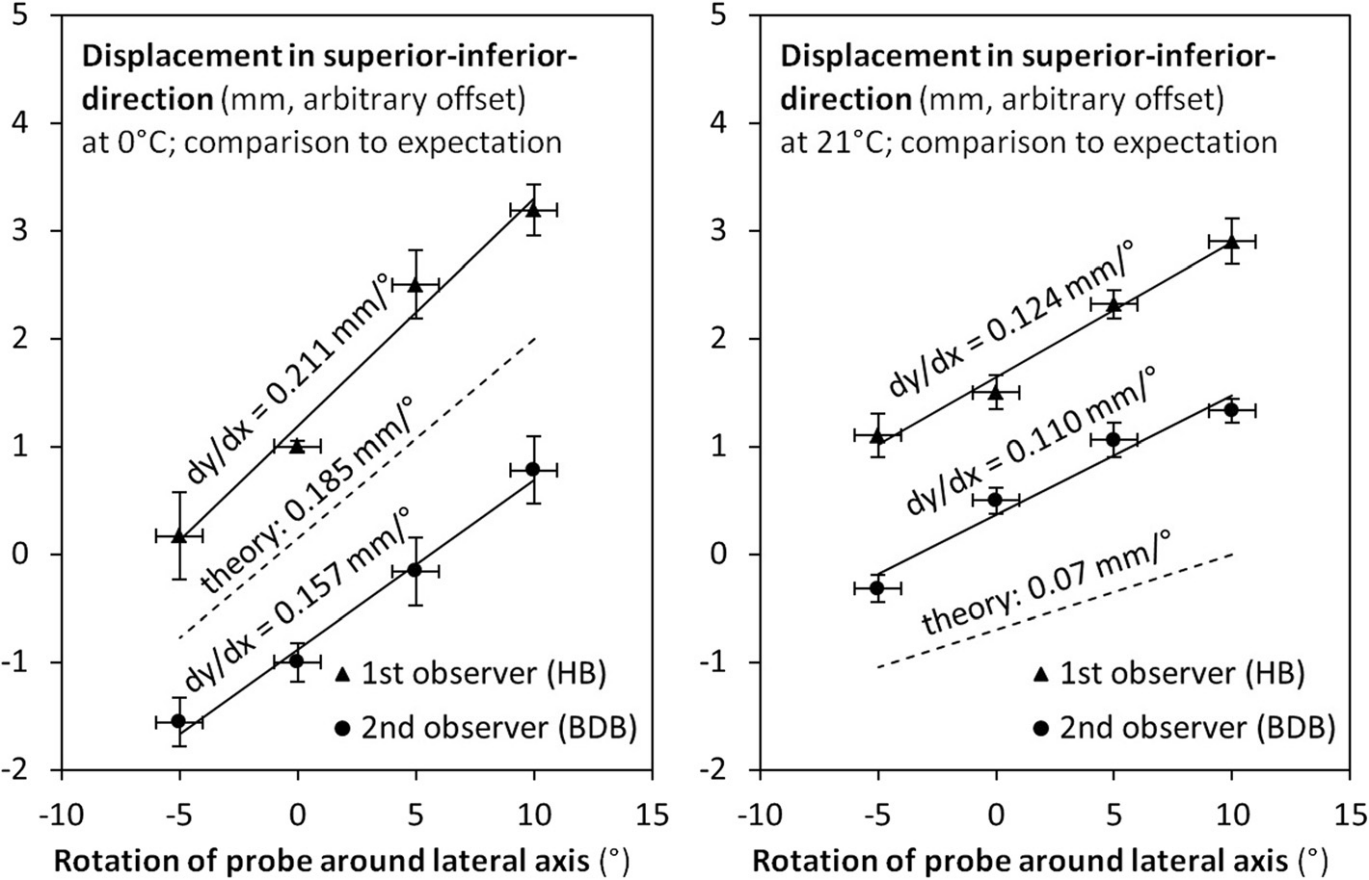
The temperature dependence is visible from the two experiments at 0 °C (left) and 21 °C (right). The lower the water temperature, the more pronounced the difference in refraction index became

During the second and third series of measurements two of the observers measured slopes dx / dα of 0.18 ± 0.04 mm/° at 0 °C, see Table 1 and Fig. 4 (left), and 0.12 ± 0.01 mm/°, respectively, at as 21.4 ± 0.4 °C (range 20.9 to 21.9), see Table 1 and Fig. 4 (right).

The speed of sound in water at 0 °C is 1,402 ms^-1^, and 1,487 ms^-1^ at 21.4 °C by Marczak’s interpolation [18]. Thus, the theoretical expectation for d(Δx/Δy) / dα was 1,540 ms^-1^ / 1,402 ms^-1^ – 1 = 0.0984 at 0 °C, and 1,540 ms^-1^ / 1,487 ms^-1^ – 1 = 0.0356 at 21.4 °C. These value times 107.5 mm (the depth Δy of the calibration feature spheroid below the surface of the phantom) yielded 10.5 mm (in units of radian) or 0.19 mm/° at 0 °C and 3.86 mm (in units of radian) or 0.07 mm/°.

During the three experiments, it was also checked whether there was a slope in lateral or vertical direction, as well. However, the only direction showing a significant trend was the longitudinal direction, the dependency in other directions was smaller by an order of magnitude or more, see Table 1.

## Discussion

Three experiments were performed. During the first experiment, a systematic dependency of the calibration of a 3D-US system in superior-inferior direction on the orientation of the ultrasound probe in the sagittal plane was detected. This linear dependency was qualitatively explainable by the refraction of ultrasound waves propagating in the sagittal plane through the water-phantom interface. During the second experiment, water was replaced by a mixture of ice and water with a physically defined melting temperature of 0 °C. The slope of the linear dependency between calibration result and probe orientation became steeper, as would be expected from a lower speed of sound in cooler water, leading to an increased difference in speed of sound versus inside the phantom. The speed of sound in Zerdine is reported to be 1,540 m/s at 22 °C by the manufacturer. The dependence of this figure on temperature is not known. However, the mass of the phantom, and presumably its specific heat capacity, is much larger than that of the thin layer of water at its top. Hence, it may be assumed to remain at room temperature during the measurement, and room temperature at our lab is well consistent with 22 °C.

Quantitatively, the second experiment yielded a slope of 0.18 ± 0.04 mm/° in full agreement with the theoretical expectation of 0.19 mm/°. There were only two experimenters, and the inter-observer spread was larger than during the first series of measurements.

During the third experiment, water, phantom and apparatus were thermalized at room temperature of 21 °C. The results of the third experiment confirmed the slope of about 0.11 to 0.12 mm/° from the first experiment. Here, the theoretical expectation of 0.07 mm/° was not met. The difference, however, was not significant (p = 0.09 by the two-tailed t-test).

Nonetheless, the dependence of the effect on the water temperature is a strong indicator for a local physical effect. This fact rules out other explanations such as under-compensation by the 3D tracking system, for example (an alternative hypothesis could have been that the tilt of the ultrasound probe had not been registered properly by the infrared camera).

So far, the effect has not been confirmed in clinical routine at our institution. There, the ultrasound probe is tilted in place rather than swept along, and the probe is firmly pressed against the patients’ skin to improve image quality, and the deformable tissue follows the probe’s curvature. Thus, the gel layer between probe and patients is thin and flat rather than wedge shaped. As a result, refraction effects are not expected, and noise from other sources of error is probably dominant. However, speed of sound will somewhat vary between tissues, and image deformations due to refraction will always be present to some degree. Still, without further investigation, additional care in accounting for refraction effects at the moment is only warranted during calibration. There are two ways to circumvent refractive effects. Either the ultrasound probe needs to be held upright during calibration, or a coupling medium needs to be used which has the same speed of sound in Zerdine. If ultrasound gel is not convenient, at 20 ± 0.75 °C a mixture of 9.5 ± 0.25 vol % ethanol in distilled water has the required speed of sound of 1,540 ± 1.5 m/s [19].

The latter solution may also be useful in other geometries of the ultrasound probe, for example, for phased arrays where some of the ray bundles enter the phantom at an angle, no matter the orientation of the ultrasound probe.

## Conclusions

The surface refraction of sound waves my affect the calibration of three-dimensional ultrasound. The temperature dependence of the effect rules out alternative explanations for the observed shifts in calibration. At room temperature and for a structure that is 10 cm below the water-phantom interface, a tilt of the ultrasound probe of 10° may result in a position reading that is off by more than half of a millimeter. Such an error would be of the same order of magnitude as other errors typically encountered during the calibration of a 3D-US system [11]. While this particular surface effect is not expected to be present in patients (where the skin follows the curvature of the ultrasound probe), an inadvertent offset in calibration can affect patient positioning just the same. Hence, if tap water or distilled water is used for calibration, care must be taken not to tilt the ultrasound probe.
